# Special Issue “Adipose Tissue and Gene Expression”

**DOI:** 10.3390/ijms27031479

**Published:** 2026-02-02

**Authors:** Margherita Maffei, Gaia Scabia

**Affiliations:** CNR, Institute of Clinical Physiology, 56124 Pisa, Italy

Since the recognition of white adipose tissue (WAT) as an endocrine organ rather than merely an energy reservoir, interest in this complex ensemble of cell types, characterized by extraordinary and, in many respects, unique plasticity, has steadily increased [1]. Beyond its initially recognized role in the regulation of long-term energy homeostasis, it has become increasingly clear that WAT profoundly influences fertility, immune responses, glucose metabolism, bone remodeling, and blood pressure regulation [2,3]. It is now reasonable to conclude that virtually no physiological function is exempt from WAT control, a concept closely linked to the central role of energy availability in individual survival. Concomitantly, WAT itself is highly responsive to a wide range of stimuli, acting as a dynamic sensor of environmental and systemic changes [4]. The strong response to this Special Issue underscores the scientific relevance and broad interest surrounding this field. The original aim has been fully achieved, bringing together studies that highlight both the multifaceted nature of WAT and its remarkable plasticity—an adaptive feature fundamentally driven by changes in gene-expression regulation.

Indeed, although at first glance the nine studies featured in this issue may appear to have little in common, they share a clear mechanistic core centered on lipid metabolic pathways and gene-expression programs that shape cellular growth and function. Together, they span species ranging from closely related mammals to a phylogenetically distant invertebrate (*Bombyx mori*); address diverse conditions, including COVID-19, mastitis, obesity, prostate cancer, and diabetes; and dissect regulatory layers encompassing nutritional cues, microRNA-mediated control, transcriptional networks, and RNA-splicing mechanisms.

Regarding gene expression, a fil rouge running through most of the studies, Pérez- Gómez J.M. and colleagues (Contribution 1) focus on the importance of optimizing molecular tools to enhance the reliability and precision of WAT gene-expression analyses. The authors applied two widely used gene-expression methodologies in combination with multiple analytical algorithms to validate a panel of previously reported internal reference genes (IRGs) in two independent cohorts of periprostatic WAT, a specialized adipose depot positioned at the pathophysiological interface between obesity and prostate cancer. They identify *LRP10*, *PGK1*, and *RPLP0* as the genes with the highest stability to be employed in gene expression studies involving human periprostatic WAT.

A considerable number of the studies rely on omic approaches to unravel how metabolic pathways and gene regulation shape tissue physiology across species. Poklukar K. and collaborators (Contribution 2) show that a protein-restricted diet in pigs affects WAT composition and transcriptomic responses in ways that depend on rearing conditions. Their results highlight how nutrition and environment converge on lipid synthesis, stress responses, and immune pathways, further emphasizing the plasticity of adipose biology.

A similar metabolic sensitivity emerges in dairy cows. Zhou B. and co-authors (Contribution 3) investigated the relationship between fatty acid metabolism and clinical mastitis (CM) through the identification and characterization of differentially expressed proteins in healthy and CM-affected animals. They found that the enzyme ACOT7 is positively correlated with CM onset and progression, linking long-chain fatty acid elongation and unsaturated lipid biosynthesis to inflammatory pathology.

Alazaabi M. and co-authors (Contribution 4) provide a developmental view of adipocyte formation. Using mouse embryonic stem cells, they mapped the transcriptional choreography underlying adipogenesis and, through a Robust Rank Aggregation approach, identified Atf5, Ccnd1, and Nr4a1 as potential fine regulators of this process.

Two contributions illustrate the importance of microRNAs as fine-tuners of adipose cell biology. In obese rabbits, Shao J. and co-authors (Contribution 5) show that miR-let-7a-5p dampens PI3K–AKT–mTOR signaling, acting as a modulator of lipid accumulation. Using primary rabbit white adipocytes, Sun W. and collaborators (Contribution 6) demonstrate that miR-889-3p facilitates the differentiation toward a brown-adipocyte phenotype and promotes the expression of thermogenic markers, likely through the inhibition of the DNA-binding protein SON.

Using genetic manipulation and molecular assays, Qin X. and co-authors (Contribution 7) shift the attention to invertebrates and demonstrated that the domestication of *Bombyx mori*, and the improvement of its economic traits for the silk market, are associated with the loss of a transposable element upstream of *Mlx*. Silencing *Mlx* decreases the expression of glucose-metabolism and lipogenic genes, whereas its overexpression in the silkworm fat body increases larval body size, cocoon-shell weight, and reproductive output.

Gene expression dynamics in adipose tissue could have a broad clinical implication in diverse pathologies. Salazar M. and co-authors (Contribution 8) demonstrate how obesity, aging, and metabolic history influence viral susceptibility at the tissue level. By profiling the differential expression of SARS-CoV-2 cell infection mediators (ACE2, TMPRSS2, ADAM17, and NRP1) in different adipose depots from middle-aged and older women with distinct metabolic backgrounds, they show that visceral adipose tissue may act both as a sentinel and a source of vulnerability in the context of COVID-19 pathogenesis, in a manner dependent on other risk factors such as obesity and aging. This is relevant as obesity and aging lead to a significantly elevated risk of developing severe and fatal forms of the disease [5,6]. Lui A. and co-authors (Contribution 9) uncover how alternative splicing affects adipocyte dysfunction in type 2 diabetes. Working with human-visceral-adipose-derived stem cells, the authors reveal that in the context of diabetes, WAT expresses a truncated, alternatively spliced form of sortilin that interferes with GLUT4 trafficking and impairs glucose uptake. They further show that GLP-1 can suppress this aberrant isoform by modulating TRA2B-dependent splicing, a regulatory mechanism that opens a therapeutic window for rescuing metabolic function in insulin-resistant adipose tissue.

Taken together, these studies form a coherent narrative: despite differences in species, techniques, and biological questions, they converge on the same underlying principle, i.e., the metabolic and regulatory versatility of adipose tissue. Whether shaping viral entry, responding to diet and environment, driving inflammation, enabling developmental transitions, directing domestication, or fine-tuning lipid storage and thermogenesis, adipose tissue consistently emerges as a central node of biological regulation.

We sincerely thank all the authors, reviewers, and editors whose collective efforts have shaped this Special Issue. By collating diverse models and cross-species perspectives, their contributions demonstrate the strong and continuing interest in this topic and exemplify the collaborative and integrative spirit needed to advance our understanding of adipose biology in health and disease.
